# County-Level Pregnancy Criminalization Rates and Prenatal Care Initiation and Utilization in Alabama, 2014–2022

**DOI:** 10.1016/j.whi.2026.03.004

**Published:** 2026-04-16

**Authors:** Taylor Riley, Jaquelyn L. Jahn, Maeve E. Wallace

**Affiliations:** a Carolina Population Center, University of North Carolina at Chapel Hill, Chapel Hill, North Carolina; b The Ubuntu Center on Racism, Global Movements, and Population Health Equity, Dornsife School of Public Health, Drexel University, Philadelphia, Pennsylvania; c Department of Epidemiology and Biostatistics, Dornsife School of Public Health, Drexel University, Philadelphia, Pennsylvania; d Department of Health Promotion Sciences, Mel & Enid Zuckerman College of Public Health, University of Arizona, Tucson, Arizona

## Abstract

**Objective:**

We investigated whether higher rates of county-level pregnancy-related arrests were associated with delayed and inadequate prenatal care among births in Alabama (2014–2022).

**Methods:**

We used restricted-use natality data from the National Center for Health Statistics and data on annual pregnancy-related arrests by county from Pregnancy Justice. We fit log Poisson generalized estimating equation models with cluster robust standard errors to estimate the relative risk and 95% confidence intervals (CIs), stratified by race and ethnicity, and adjusted for individual- and county-level confounders.

**Results:**

Fully adjusted models suggest individuals living in counties with higher pregnancy criminalization rates were moderately more likely to delay prenatal care initiation (1.11, 95% CI [1.08, 1.14]) and receive inadequate prenatal care (1.05, 95% CI [1.03, 1.06]) compared with those living in a county with no arrests. We found positive associations among non-Hispanic white and Latina/Hispanic women, and null or slightly protective associations among non-Hispanic Black women.

**Conclusions:**

These findings suggest pregnancy criminalization has an indirect chilling impact on prenatal health care access. The rise in the criminalization and surveillance of pregnancy, particularly in a post-*Dobbs* context, requires evidence-based policy responses that support maternal health and reproductive autonomy.

The criminalization of pregnancy-related behaviors and pregnancy outcomes is a growing public health and legal issue in the United States. There has been a marked rise in pregnancy criminalization, which occurs when an individual is charged with a crime related to their pregnancy, pregnancy loss, or birth (Bach & Wasilczuk, 2024; Kavattur et al., 2023; Paltrow & Flavin, 2013). In these cases, pregnancy transforms otherwise legal conduct or behaviors into a crime (Bridges, 2020). Most pregnancy criminalization cases involve allegations of prenatal substance use and disproportionately impact low-income women (Kavattur et al., 2023). A growing number of cases involve prosecution of miscarriage and stillbirth where women are charged with crimes such as “abuse of a corpse” or murder (Bach & Wasilczuk, 2024; Goodwin, 2020; Kavattur et al., 2023; Pregnancy Justice, 2025). Increasingly, these cases can be highly publicized and can inhibit care-seeking behaviors because they reinforce a public understanding that, in the words of legal scholar Michele Goodwin, “law enforcement interests may supersede or even outweigh the goals of medical care and treatment” (Goodwin, 2017). This accelerating trend in pregnancy criminalization is fueled by the increasing adoption of fetal personhood laws and ideology that aims to give fertilized eggs, embryos, and fetuses legal rights, often subordinating the rights of the pregnant person under the rationale of protecting the fetus from suspected harm (McGovern et al., 2025; Pregnancy Justice, 2024b; Riley & Hassan, 2025).

Pregnancy criminalization often begins in medical settings where hospital staff or social workers report their pregnant patients to law enforcement (Bach, 2022; Goodwin, 2020; Howard, 2024; Kavattur et al., 2023; Paltrow & Flavin, 2013; Roberts, 1997). This turns medical institutions into sites of criminalization and surveillance (Brayne, 2014; Goodwin, 2017). Such transformations can increase fear of legal consequences among pregnant individuals, particularly those from marginalized communities, and lead to avoiding or delaying prenatal care, ultimately undermining both maternal and infant health (Boone, 2022; Goodwin, 2017). Many cases of pregnancy criminalization result from health care providers drug testing pregnant patients and newborns, which can contribute to mistrust and reduce engagement in prenatal care (Faherty et al., 2020; Stone, 2015). The widespread practice of testing for and punishing prenatal substance use, which was established during the “War on Drugs” and disproportionately targeted low-income Black communities, was then extended to target poor white communities in the methamphetamine and opioid epidemics (Bridges, 2020; Kavattur et al., 2023; Paltrow & Flavin, 2013; Roberts, 1997). Substantial research links punitive prenatal substance use policies with lower prenatal care utilization among individuals who use substances and also at the population level (Austin et al., 2022; Boone & McMichael, 2021; Herbolsheimer & Burge, 2022; Meinhofer et al., 2022). Population-level maternal health impacts have also been identified from other forms of policing (e.g., fatal police violence, proactive policing tactics such as frequent officer-initiated stop-and-frisk) (Goin et al., 2021; Hardeman et al., 2021; Jahn et al., 2023; Riley et al., 2024). However, no studies, to our knowledge, have examined the population health impacts of living in environments where pregnancy conduct and outcomes are increasingly criminalized.

Although there is widespread concern about this emerging legal trend among legal scholars, public health professionals, and reproductive justice advocates, there is limited empirical data on the impacts of pregnancy criminalization on pregnancy-related health care and outcomes. This study seeks to address this gap by examining the relationship between county-level pregnancy criminalization arrest rates and individual-level prenatal care initiation and utilization. We leverage individual-level natality data and county-level pregnancy criminalization cases from Pregnancy Justice, which has compiled the most comprehensive database of documented pregnancy-related arrests to date (Kavattur et al., 2023). Drawing on the substantial body of literature linking policing with health care avoidance (Alang et al., 2023; Alang et al 2021; Brayne, 2014; Kerrison & Sewell, 2020; Sewell et al., 2021), we hypothesize that individuals residing in counties with higher levels of pregnancy criminalization will initiate prenatal care later in pregnancy and attend fewer prenatal visits overall, adjusting for sociodemographic and health care access factors. Additionally, we hypothesize this will disproportionately affect racially minoritized populations because of the existing disproportionate criminalization and surveillance of racially minoritized communities. Based on legal and journalistic research (Bach, 2022; Howard, 2024; Martin, 2015; National Academies of Sciences, Engineering, and Medicine, 2023), we conceptualized that pregnant people can become aware of (i.e., “exposed to”) these pregnancy-related arrests through word of mouth (e.g., known health care providers who administer prenatal drug tests) and local media coverage, which aligns with how case data were collected.

## Materials and Methods

### Study Context, Data, and Population

We conducted this multilevel analysis using two primary data sources: individual-level restricted-use natality data from the National Center for Health Statistics (NCHS) and county-level annual data on pregnancy-related arrests from the most comprehensive database available from the legal organization Pregnancy Justice (further details below). We restricted the analysis to Alabama for several reasons. First, Alabama is the leading state in the country for criminalizing pregnancy outcomes and conduct, accounting for nearly half (47%) of all documented pregnancy-related arrests before the Supreme Court *Dobbs v. Jackson Women’s Health Organization* decision (Kavattur et al., 2023). The state’s laws and judicial decisions have expanded the definition of “child” to include fetuses (Howard, 2024; Pregnancy Justice, 2024b). As a result, most documented pregnancy-related arrests in Alabama involve pregnant individuals prosecuted for substance use under the crime of “chemical endangerment of a child,” even in the absence of harm to the fetus (Howard, 2024; Kavattur et al., 2023). Because most documented Alabama cases occurred under this chemical endangerment charge, researchers were able to more easily identify pregnancy criminalization cases through public records requests compared with other state settings (Howard, 2024; Kavattur et al., 2023; ProPublica, 2015). Alabama also has had more robust data collection due to combined legal, academic, and journalistic research efforts (Howard, 2024; Kavattur et al., 2023; ProPublica, 2015). Therefore, although this database is an undercount of pregnancy criminalization cases due to difficulty in data collection, Alabama has more complete case ascertainment of pregnancy-related arrests compared with other states. Last, Alabama consistently performs worse than the national average on maternal and infant health outcomes, including prenatal care access, and there is a critical need to examine the contextual determinants of the state’s poor maternal health (Collins et al., 2024).

The study population included all singleton live births among Alabama residents from January 1, 2014, to May 31, 2022 (*n* = 474,232). This study period maximizes data quality and availability. The start date aligns with Alabama’s adoption of the NCHS revised birth certificate that improved data quality (Division of Statistical Analysis, Center for Health Statistics, 2019) and the end date aligns with the availability of pregnancy-related arrest data before the 2022 *Dobbs* decision, which overturned the constitutional right to abortion. Of note, NCHS natality data do not include information on gender identity and we use the term “women” in accordance with the source data but recognize that not all people who give birth identify as a woman (i.e., some are transgender men and gender nonconforming people).

### Exposure

Our exposure was documented pregnancy-related arrests at the county level, which we extracted from the Pregnancy Justice database. Cases were included in the database if information on the case’s name, year, county, and state were available and if the individual faced an arrest, arrest warrant, or harsher criminal penalties due to their pregnancy (Kavattur et al., 2023). Researchers identified cases through a variety of sources, including legal databases (e.g., WestLaw, LexisNexis, Bloomberg Law), Google news alerts, police blotters, media coverage, public records requests, court dockets, and referrals from attorneys, journalists, or from the organization’s own involvement in cases (Howard, 2024; Kavattur et al., 2023; ProPublica, 2015). Cases that met inclusion criteria were qualitatively coded using a standardized protocol, which was iteratively refined to maximize intercoder reliability, for information related to demographic characteristics, context and grounds for arrest, actors involved in instigation of arrest, procedural characteristics, and pregnancy outcomes (Kavattur et al., 2023). This information was collected and coded from January 1, 2006, to June 23, 2022, the day before the *Dobbs* decision. Due to confidentiality protections, we received aggregated data on the annual number of documented pregnancy-related arrests in each county. Further details on data collection are published elsewhere (Kavattur et al., 2023).

We calculated county- and time-specific pregnancy criminalization rates as the number of documented pregnancy-related arrests occurring in the maternal county of residence during the 2 years prior to birth divided by the number of live births during the same time in that county. We examined rates for the cumulative two calendar years prior to the birth year to ensure adequate pre-pregnancy exposure for all births given the annual exposure data (e.g., the exposure rate for a birth in 2020 was calculated with the total number of pregnancy-related arrests and births occurring in that county in 2018 and 2019). We calculated time-varying categories of arrest rates based on within-year averages to account for high zero counts, a long right tail in the rate distribution, and rate changes over time (No arrests [0 arrests per 100,000 births]; Medium [>0 to annual average arrest rate]; and Highest [> average annual arrest rate]).

### Outcome

The two primary individual-level outcomes in the natality data were delayed prenatal care initiation and inadequate prenatal care utilization. Delayed prenatal care initiation was operationalized as late or no prenatal care (late defined as beginning care in the third trimester) (Osterman & Martin, 2023). To measure prenatal care utilization, we used the Adequacy of Prenatal Care Utilization Index, which provides an appropriate index for evaluating the extent of prenatal care utilization, particularly after care is initiated (Alexander & Kotelchuck, 1996; Martin & Osterman, 2023). The index uses birth record data on the timing of initiation of prenatal care, gestational duration, and total number of prenatal care visits to categorize the adequacy of prenatal care into intensive, adequate, intermediate, and inadequate. Similar to other studies (Choi et al., 2025), we categorized our final binary outcome as inadequate/intermediate (referred to as inadequate hereafter) versus adequate and intensive care. Inadequate care was defined as receiving care after the fourth gestational month or receiving less than half of the appropriate number of visits based on gestational duration. Intermediate care was defined as receiving care in the first 4 gestational months but with 50–79% of the recommended number of visits (Alexander & Kotelchuck, 1996; Martin & Osterman, 2023).

### Covariates

Individual-level variables from the natality data included maternal age (<20, 20–29, 30–39, 40+), self-reported race and ethnicity (non-Hispanic [NH] American Indian/Alaska Native, NH Asian, NH Black, Latina/Hispanic, NH multiracial, and NH white), education (high school or less, some college, bachelor’s degree or more), Medicaid enrollment, parity, marital status, and nativity. To account for county-level differences in drivers of health care access and criminalization, we included the following county-level covariates: rurality based on the NCHS Urban-Rural classification scheme for counties; economic and racialized segregation as measured by the index of concentration at the extremes (ICE_race-income_) using the American Community Survey 2014–2018 and 2018–2022 5-year estimates, which quantifies the concentration of a county population in the most deprived group (low income and Black) to the most privileged group (high income and white) (Krieger et al., 2016); a proxy for prenatal care availability and access, measured as the number of obstetrician-gynecologists (OBGYNs) per 100,000 births in each county from the Area Health Resources File of the U.S. Department of Health and Human Services; and annual jail incarceration rates of adults 15–64 years old from the Vera Institute of Justice.

### Statistical Analysis

We first descriptively examined individual birth and county-level characteristics across pregnancy criminalization rates and our two prenatal care outcomes. We fit log Poisson generalized estimating equation models with an independent working correlation structure and cluster robust standard errors to estimate the relative risk (RR) and 95% confidence intervals (CIs) of each outcome across categories of pregnancy criminalization rates (Yelland et al., 2011). We fit crude and adjusted models with individual and county-level covariates described above, in addition to year fixed effects and a dummy variable indicating before versus during the COVID-19 pandemic (coded as starting March 1, 2020), which impacted both criminal legal systems and prenatal health care access (Jahn et al., 2022; Kotlar et al., 2021). We performed effect modification analyses by maternal race and ethnicity among racialized groups with adequate sample sizes (NH Black, Latina, and NH white).

We performed several sensitivity analyses to validate our findings. First, we examined exposure rates in the calendar year before birth (compared with 2 years before birth) to assess if there were any differences in model estimates with a closer temporal exposure. Second, while we conceptualized the county-level confounders (racialized economic segregation, OBGYN availability, and jail incarceration) as upstream of both pregnancy criminalization and prenatal care access, it is possible increased pregnancy criminalization could impact these county-level measures (e.g., increased criminalization pushes OBGYNs to relocate or increases jail incarceration rates), so we ran adjusted models just adjusting for individual confounders and removed the county-level confounders. Finally, while the pregnancy-related arrest data source is the most comprehensive information on documented cases available, these are likely an undercount and therefore there is exposure misclassification (underestimation) present. Of note, though, because the data collection relied on media reports and public records, pregnancy-related arrests not documented were not covered by media or known by local advocates, and thus there was likely limited knowledge of these arrests in the community (i.e., few people were “exposed” to these arrests via our hypothesized exposure pathways of media and word of mouth). However, to address exposure misclassification if there was some systematic reason why some counties had documented arrests and others did not, we performed a sensitivity analysis restricting the analysis to counties that reported any arrests during the study period and removing counties with no documented arrests. Analyses were conducted in R version 4.4.2 and this de-identified secondary data analysis was determined exempt by the University of North Carolina at Chapel Hill Institutional Review Board.

## Results

From 2014 to 2022, 474,232 births occurred among individuals residing in Alabama; 8% of these individuals initiated prenatal care late or not at all and 25% received inadequate prenatal care (Table 1). There were 447 documented pregnancy-related arrests during the study period, with average annual county-level rates ranging from 58 to 104 pregnancy-related arrests per 100,000 births. Of the 67 counties in Alabama, at least one pregnancy-related arrest was documented in 43 of the counties (64%) during the study period. Counties with the highest pregnancy criminalization rates were also more likely to be in the highest ICE_race-income_ quintile (most privileged) and have higher jail incarceration rates. More than a quarter of white (26%) and Latina (30%) women lived in counties with the highest rates of pregnancy criminalization, but a smaller proportion of Black women (12%) lived in these counties. Compared with those with timely prenatal care initiation, women with delayed prenatal care initiation were, on average, younger, identified as Latina, had a high school education or less, had one or more children, were not married, were born outside the United States, had Medicaid coverage, and resided in a rural county with lower OBGYN availability and higher jail incarceration and pregnancy criminalization rates (Table 2). Similar individual-level patterns were observed with prenatal care utilization, with the addition of a higher proportion of individuals with inadequate prenatal care identifying as Black compared with those with adequate prenatal care (Table 2).

Fully adjusted models among all births suggest, on average, higher county-level pregnancy criminalization rates were associated with delayed and inadequate prenatal care while adjusting for individual and county-level confounders (Table 3). Women living in counties with the medium or highest levels of county-level pregnancy criminalization rates were, respectively, 6% and 11% more likely to initiate prenatal care late or not at all compared with those living in counties with no arrests (None vs. Medium, adjusted RR [aRR], 1.06; 95% CI [1.04, 1.09]; None vs. Highest, aRR, 1.11; 95% CI [1.08, 1.14]). The relationship between pregnancy criminalization and inadequate prenatal care utilization was moderately positive when comparing None versus Medium counties (aRR, 1.05; 95% CI [1.03, 1.06]) and crossed the null for None versus Highest counties (aRR: 1.01; 95% CI [.99, 1.02). For both outcomes, the fully adjusted models were generally similar or more attenuated towards the null compared to the models only adjusting for individual-level covariates (Appendix Table 1).

In the racial and ethnic group stratified models, we found positive associations between county-level pregnancy criminalization rates and late/no prenatal care initiation among white and Latina women, but a null or inverse association among Black women (Table 3). The effect size was substantially larger among Latina women, where, for example, the risk of late or no prenatal care was 18% higher among Latina women living in counties with higher county-level pregnancy-related arrest rates compared with no arrests (None vs. Medium, aRR, 1.18; 95% CI [1.12, 1.24]; None vs. Highest, aRR, 1.18; 95% CI [1.13, 1.24]). Regarding inadequate prenatal care utilization, we observed positive associations among white women (None vs. Medium, aRR, 1.08; 95% CI [1.06, 1.10]; None vs. Highest, aRR, 1.04; 95% CI [1.02, 1.06]). Conversely, we found a null or modest inverse association among Black women (None vs. Medium, aRR, 1.01; 95% CI [.98, 1.03]; None vs. Highest, aRR, .93; 95% CI [.90, .96]) and Latina women (None vs. Medium, aRR, 1.02; 95% CI [.99, 1.06]; None vs. Highest, aRR, .97; 95% CI [.93, .99]). In sensitivity analyses additionally adjusting for county-level differences in the Latina population, the direction of associations remained similar and effect sizes were smaller or attenuated to the null among Latina women (Appendix Table 2).

Sensitivity analyses with exposure rates in the calendar year before birth (compared with 2 years prior) were substantively similar overall and across racial and ethnic groups (Appendix Table 3). Additionally, in models removing counties with no documented arrests during the study period, we observed similar results, with the exception of slightly smaller effect sizes among Latina women (Appendix Table 4).

## Discussion

Our study sought to elucidate the multilevel relationship between pregnancy criminalization and prenatal care initiation and utilization in Alabama over a nearly 9-year study period. We found that higher county pregnancy criminalization rates were associated with greater risk of delayed and inadequate prenatal care overall and particularly among white and Latina women. These findings suggest pregnancy criminalization has indirect and chilling effects on prenatal care. Notably, fear and avoidance of pregnancy-related care is likely to increase as the threat of criminalization becomes more widespread following the Supreme Court *Dobbs v. Jackson Women’s Health Organization* decision, which overturned the constitutional right to abortion and paved the way for more legal surveillance of pregnancy (Harris, 2024; McGovern et al., 2025; Paltrow et al., 2022; Riley & Hassan, 2025). In the 2 years following *Dobbs*, there were at least 412 cases charging pregnant people with crimes related to pregnancy, pregnancy loss, or birth, and these are some of the highest annual numbers documented since 1973 (Bach & Wasilczuk, 2024; Pregnancy Justice, 2025).

Although pregnancy criminalization has increased rapidly in the past two decades (Bach & Wasilczuk, 2024; Kavattur et al., 2023), there is limited empirical research that has examined the implications for prenatal care outcomes and inequities. It is widely accepted medical consensus that timely and adequate prenatal care improves maternal and infant health outcomes (Gortmaker, 1979; Partridge et al., 2012). Although many of these prosecutions, related fetal personhood laws, and judicial decisions use the rationale of promoting fetal health, our findings suggest this legal trend is undermining both fetal and maternal health by reducing prenatal health care access. Our findings also align with other research on punitive prenatal substance use laws that find criminalizing pregnancy-related conduct worsens population-level maternal and infant health (Boone & McMichael, 2021; Herbolsheimer & Burge, 2022; Meinhofer et al., 2022). These studies, combined with ours, illuminate how the harms of criminalizing pregnancy conduct extend beyond those directly criminalized and can worsen health care access and outcomes in surrounding communities. This bolsters the broader literature that documents how even indirect exposure to criminalization and punitive surveillance can lead to fear of institutions, medical mistrust, and worse health outcomes (Alang et al., 2021; 2023; Brayne, 2014; Kerrison & Sewell, 2020; Novak et al., 2017; Sewell et al., 2021).

Our findings from the race and ethnicity stratified models were notable. First, we found larger effect sizes among Latina women. Nationally, Latina and Black women are less likely to access timely prenatal care and receive adequate prenatal care relative to white women (Choi et al., 2025), a pattern we also observed in Alabama. In Alabama, 41.5% of Latina women of reproductive age are uninsured, which is well above the national average (26.5%) (Clark & Smith, 2023). Our study also found that Latina women were, on average, exposed to higher county-level rates of pregnancy criminalization in Alabama. We hypothesize that pregnancy criminalization may be operating as a psychosocial stressor, similar to other forms of criminalization. For instance, immigration raids are associated with acute increases in adverse birth outcomes among both U.S.-born and immigrant Latina women (Novak et al., 2017; Ro et al., 2020). The risk of possible criminalization during pregnancy may add to immigration-related concerns that deter prenatal care among immigrant Latinas, those in mixed-status households, and those at risk of being perceived as undocumented (Saadi et al., 2020; White et al., 2014). Additionally, there is substantial heterogeneity in ethnicity and immigration histories within Latina populations that we were unable to examine in this study. Future research should disaggregate to further explore the relationship between criminalization and reproductive health among Latina women.

Second, contrary to our hypothesis, we observed null or slightly protective associations between county-level pregnancy criminalization and prenatal care outcomes among Black women and moderately higher risk among white women. Notably, Black women in Alabama were less likely to reside in counties with the highest rates of pregnancy criminalization, while white women were more likely to reside in these counties. Most pregnancy-related arrests have been among white women, largely reflecting the fact that enforcement has been strongest in these counties with majority white populations (Pregnancy Justice, 2024a). The differential prosecutorial discretion for enforcement by county, combined with the substantial residential segregation in Alabama (Menendian et al., 2021), likely impacts our hypothesized exposure mechanisms whereby Black women may be less aware of these arrests. It is important to note that the racist carceral approaches to prenatal substance use that have targeted low-income Black women are now being more broadly applied to low-income white communities impacted by substance use (Bridges, 2020; Goodwin, 2017; Kavattur et al., 2023; Roberts, 1997). The interplay of geography, race and racism, and poverty continue to shape pregnancy criminalization, and future research should investigate the intersectional public health impacts of pregnancy criminalization by race, class, and place.

### Limitations

This study has important limitations to consider. First, the pregnancy-related arrest data, although the best available, were undercounted due to difficulty in data collection. This non-differential misclassification of exposure (i.e., underestimation of arrests does not differ systematically by prenatal care outcomes) likely biases our estimates toward the null, but future research with more complete data would improve exposure measurement. However, we hypothesized that individuals in the community were likely exposed to information about pregnancy-related arrests through word of mouth and/or local media coverage, so it is likely that if a pregnancy-related arrest was not documented from news alerts, media coverage, and public data requests, then it is unlikely broader communities were aware of this arrest. Additionally, due to confidentiality protections, we did not have individual-level information (e.g., demographics, type of charge) on pregnancy-related arrests. These arrests also likely represent the “tip of the iceberg” of surveillance and criminalization of pregnancy, because many more pregnant individuals may have experienced what journalists have referred to as “the chemical-endangerment version of stop-and-frisk” of nonconsensual drug testing or invasive child welfare investigations that did not culminate in an arrest, which are not captured in our exposure measure (Martin, 2015). Second, although our hypothesized exposure mechanisms drew from established research on environments of criminalization and health care access (Alang et al., 2023; Bach, 2022; Brayne, 2014; Howard, 2024; Kerrison & Sewell, 2020; Sewell et al., 2021), we were unable to measure mechanisms of media exposure, word of mouth, or individual awareness of arrests in this study. Future research should examine how this information is spread in communities to inform public health interventions and messaging. Third, although the county level is relevant for how and where cases are generally prosecuted, it is possible that the hypothesized pathways of exposure might not be captured well at the county level, and spillover effects across counties is likely. Fourth, the Alabama context may not be generalizable to other states with different trends in pregnancy criminalization and prenatal care. Fifth, birth records data lack robust information about individual socioeconomic status, which is an important factor in pregnancy criminalization. Future research should examine this relationship among economically marginalized women specifically. Last, due to limitations in available data, our exposure rate used live births as a denominator, but these arrests can occur in situations involving non-live births. However, we believe the degree of bias to be minimal because the vast majority of pregnancy-related arrests during this period in Alabama involved prenatal substance use and a live birth (Kavattur et al., 2023).

### Implications for Policy and Practice

Our findings linking pregnancy criminalization arrest rates to delayed and inadequate prenatal care are consequential in a post-*Dobbs* context in which an increasing number of local prosecutorial decisions and state laws are elevating fetal rights and, as a result, actively undermining maternal and infant health. This study suggests that pregnancy criminalization affects not just the individuals arrested, who undergo traumatic and harmful experiences with long-lasting health and economic impacts (Bach, 2022; Goodwin, 2017; Howard, 2024; Martin, 2015), but that the harms extend to entire communities and have negative implications for maternal health care access. Delayed and inadequate prenatal care is preventable, and our study points to intervenable policies and practices that could improve prenatal care access. Effective public health and clinical strategies could take a reproductive justice approach that holistically recognizes the rights to have children, not have children, and raise children in safe communities free from state interference and harm (Crear-Perry et al., 2021; SisterSong Women of Color Reproductive Justice Collective, n.d.). Specifically, comprehensive policy solutions that aim to improve trust in health care by addressing the interconnectedness of criminalizing abortion, pregnancy loss, and prenatal substance use—all fueled by fetal personhood—could help improve maternal health care. For example, this could include health care systems and practitioners ending the practices that fuel pregnancy criminalization, including drug testing (Roberts et al., 2024), and embracing policies that prevent other forms criminalization that occur in health care settings, including immigration criminalization.

## Supplementary Material

1

Supplementary Data

Supplementary data to this article can be found at https://doi.org/10.1016/j.whi.2026.03.004.

## Figures and Tables

**Table 1:** Characteristics of Births Across Categories of County-Level Pregnancy Criminalization Rates, Alabama, January 1, 2014–May 31, 2022

Maternal and County Characteristics	Pregnancy-Related Arrests Per 100,000 Births in the 2 Years Before Birth			Total
	None	Medium	Highest	
			
	(*n* = 204,652)	(*n* = 165,843)	(*n* = 103,737)	(*N* = 474,232)

Age, y, *n* (%)				
<20	15,705 (7.7)	10,934 (6.6)	8,285 (8.0)	34,924 (7.4)
20–29	121,688 (59.5)	92,562 (55.8)	61,646 (59.4)	275,896 (58.2)
30–39	63,783 (31.2)	58,892 (35.5)	32,008 (30.9)	154,683 (32.6)
40+	3,476 (1.7)	3,455 (2.1)	1,798 (1.7)	8,729 (1.8)
Race and ethnicity, *n* (%)				
American Indian/Alaska Native (non-Hispanic [NH])	533 (.3)	396 (.2)	195 (.2)	1,124 (.2)
NH Asian	2,846 (1.4)	3,657 (2.2)	1,261 (1.2)	7,764 (1.6)
NH Black	61,800 (30.2)	62,350 (37.6)	17,475 (16.8)	141,625 (29.9)
Latina/Hispanic	14,399 (7.0)	12,943 (7.8)	11,834 (11.4)	39,176 (8.3)
NH multiracial	3,056 (1.5)	2,565 (1.5)	1636 (1.6)	7,257 (1.5)
NH white	12,1975 (59.6)	83,883 (50.6)	71,304 (68.7)	277,162 (58.4)
Unknown	43 (.0)	49 (.0)	32 (.0)	124 (.0)
Education, *n* (%)				
High school or less	96,668 (47.2)	73,157 (44.1)	50,472 (48.7)	220,297 (46.5)
Some college	62,815 (30.7)	45,922 (27.7)	31,284 (30.2)	140,021 (29.5)
Bachelor’s degree or more	44,756 (21.9)	46,391 (28.0)	21,804 (21.0)	112,951 (23.8)
Missing	413 (.2)	373 (.2)	177 (.2)	963 (.2)
Parity, *n* (%)				
0	80,890 (39.5)	65,005 (39.2)	40,356 (38.9)	186,251 (39.3)
1 +	123,703 (60.4)	100,731 (60.7)	63,290 (61.0)	287,724 (60.7)
Missing	59 (.0)	107 (.1)	91 (.1)	257 (.1)
Marital status, *n* (%)				
Not married	96,921 (47.4)	79,617 (48.0)	42,256 (40.7)	218,794 (46.1)
Currently married	107,716 (52.6)	86,214 (52.0)	61,476 (59.3)	255,406 (53.9)
Missing	15 (.0)	12 (.0)	5 (.0)	32 (.0)
Nativity, *n* (%)				
U.S. born	188,128 (91.9)	148,681 (89.7)	92,112 (88.8)	428,921 (90.4)
Born outside of the United States	16,524 (8.1)	17,162 (10.3)	11,625 (11.2)	45,311 (9.6)
Medicaid, *n* (%)				
Yes	104,370 (51.0)	80,729 (48.7)	51,588 (49.7)	236,687 (49.9)
No	100,173 (48.9)	85,002 (51.3)	52,094 (50.2)	237,269 (50.0)
Missing	109 (.1)	112 (.1)	55 (.1)	276 (.1)
Rurality, maternal county of residence, *n* (%)				
Urban	132,311 (64.7)	156,272 (94.2)	77,803 (75.0)	366,386 (77.3)
Rural	72,341 (35.3)	9,571 (5.8)	25,934 (25.0)	107,846 (22.7)
Racialized economic segregation (ICE_race-income_), maternal county of residence, *n* (%)				
Quintile 1 – Most marginalized	24,520 (12.0)	4,574 (2.8)	4,184 (4.0)	33,278 (7.0)
Quintile 2	35,059 (17.1)	48,146 (29.0)	7,501 (7.2)	90,706 (19.1)
Quintile 3	47,452 (23.2)	69,539 (41.9)	16,356 (15.8)	133,347 (28.1)
Quintile 4	30,990 (15.1)	11,812 (7.1)	26,815 (25.8)	69,617 (14.7)
Quintile 5 – Most privileged	66,631 (32.6)	31,772 (19.2)	48,881 (47.1)	147,284 (31.1)
Jail incarceration rate per 100,000 residents age 15–64, maternal county of residence				
Mean (SD)	470 (204)	343 (166)	603 (264)	455 (228)
Median [Min, Max]	426 [22.0, 1460]	381 [59.3, 881]	531 [91.0, 1450]	399 [22.0, 1460]
OBGYNs per 10,000 births in maternal county of residence				
Mean (SD)	65.1 (56.8)	125 (52.5)	63.6 (31.7)	85.6 (58.3)
Median [Min, Max]	64.5 [0, 193]	128 [0, 193]	70.9 [0, 136]	90.3 [0, 193]
Initiation of prenatal care, *n* (%)				
On time	189,069 (92.4)	153,704 (92.7)	93,786 (90.4)	436,559 (92.1)
Late or none	14,609 (7.1)	11,421 (6.9)	9,317 (9.0)	35,347 (7.5)
Missing	974 (.5)	718 (.4)	634 (.6)	2,326 (.5)
Utilization of prenatal care, *n* (%)				
Adequate	151,059 (73.8)	120,761 (72.8)	75,282 (72.6)	347,102 (73.2)
Inadequate	49,471 (24.2)	41,806 (25.2)	24,852 (24.0)	116,129 (24.5)
Did not initiate prenatal care/Missing	4,122 (2.0)	3,276 (2.0)	3,603 (3.5)	11,001 (2.3)

**Table 2:** Maternal and County of Residence Characteristics by Prenatal Care Outcomes, Alabama, January 1, 2014–May 31, 2022

Maternal and County Characteristics	Outcome 1: Initiation of Prenatal Care		Outcome 2: Utilization of Prenatal Care	
	On Time	Late or No Prenatal Care	Adequate	Inadequate
	(*n* = 436,559)	(*n* = 35,347)	(*n* = 347,102)	(*n* = 116,129)

Age, y, *n* (%)				
<20	30,508 (7.0)	4,257 (12.0)	21,960 (6.3)	11,740 (10.1)
20–29	253,874 (58.2)	20,620 (58.3)	200,030 (57.6)	69,787 (60.1)
30–39	144,232 (33.0)	9,730 (27.5)	118,671 (34.2)	32,604 (28.1)
40+	7,945 (1.8)	740 (2.1)	6,441 (1.9)	1,998 (1.7)
Race and ethnicity, *n* (%)				
American Indian/Alaska Native (non-Hispanic [NH])	1,056 (.2)	66 (.2)	828 (.2)	281 (.2)
NH Asian	7,228 (1.7)	510 (1.4)	5,833 (1.7)	1,821 (1.6)
NH Black	130,037 (29.8)	108,83 (30.8)	98,977 (28.5)	39,918 (34.4)
Latina/Hispanic	29,743 (6.8)	9,144 (25.9)	20,211 (5.8)	14,210 (12.2)
NH multiracial	6,577 (1.5)	628 (1.8)	5,020 (1.4)	2,104 (1.8)
NH white	261,827 (60.0)	14,105 (39.9)	216,173 (62.3)	57,735 (49.7)
Unknown	91 (.0)	11 (.0)	60 (.0)	60 (.1)
Education, *n* (%)				
High school or less	194,588 (44.6)	24,553 (69.5)	145,169 (41.8)	66,108 (56.9)
Some college	132,196 (30.3)	7,134 (20.2)	108,065 (31.1)	30,505 (26.3)
Bachelor’s degree or more	109,188 (25.0)	3,364 (9.5)	93,543 (26.9)	19,028 (16.4)
Missing	587 (.1)	296 (.8)	325 (.1)	488 (.4)
Parity, *n* (%)				
0	174,129 (39.9)	11,322 (32.0)	141,851 (40.9)	41,381 (35.6)
1+	262,400 (60.1)	24,022 (68.0)	205,228 (59.1)	74,516 (64.2)
Missing	30 (.0)	3 (.0)	23 (.0)	232 (.2)
Marital status, *n* (%)				
Not married	195,787 (44.8)	21,901 (62.0)	145,907 (42.0)	66,144 (57.0)
Currently married	240,744 (55.1)	13,442 (38.0)	201,170 (58.0)	49,979 (43.0)
Missing	28 (.0)	4 (.0)	25 (.0)	6 (.0)
Nativity, *n* (%)				
U.S. born	401,144 (91.9)	25,758 (72.9)	322,094 (92.8)	100,672 (86.7)
Born outside of the United States	35,415 (8.1)	9,589 (27.1)	25,008 (7.2)	15,457 (13.3)
Medicaid, *n* (%)				
Yes	210,464 (48.2)	25,216 (71.3)	156,029 (45.0)	72,624 (62.5)
No	225,964 (51.8)	10,036 (28.4)	190,995 (55.0)	43,380 (37.4)
Missing	131 (.0)	95 (.3)	78 (.0)	125 (.1)
Rurality, maternal county of residence, *n* (%)				
Urban	339,262 (77.7)	25,265 (71.5)	269,542 (77.7)	89,547 (77.1)
Rural	97,297 (22.3)	10,082 (28.5)	77,560 (22.3)	26,582 (22.9)
Racialized economic segregation (ICE_race-income_), maternal county of residence, *n* (%)				
Quintile 1 – Most marginalized	30,177 (6.9)	2,895 (8.2)	23,087 (6.7)	9,418 (8.1)
Quintile 2	84,717 (19.4)	5,447 (15.4)	67,369 (19.4)	21,835 (18.8)
Quintile 3	123,201 (28.2)	9,597 (27.2)	96,597 (27.8)	34,293 (29.5)
Quintile 4	63,038 (14.4)	6,070 (17.2)	51,460 (14.8)	16,022 (13.8)
Quintile 5 – Most privileged	135,426 (31.0)	11,338 (32.1)	108,589 (31.3)	34,561 (29.8)
Jail incarceration rate per 100,000 residents aged 15–64, maternal county of residence				
Mean (SD)	452 (228)	483 (235)	452 (228)	455 (227)
Median [Min, Max]	397 [22.0, 1460]	432 [22.0, 1460]	398 [22.0, 1460]	397 [22.0, 1460]
OBGYNs per 10,000 births in, maternal county of residence				
Mean (SD)	86.0 (58.4)	81.6 (56.5)	85.7 (58.5)	86.2 (58.2)
Median [Min, Max]	90.3 [0, 193]	84.4 [0, 193]	90.3 [0, 193]	90.3 [0, 193]
Pregnancy-related arrest rates, maternal county of residence, *n* (%)				
None	189,069 (43.3)	14,609 (41.3)	151,059 (43.5)	49,471 (42.6)
Medium	153,704 (35.2)	11,421 (32.3)	120,761 (34.8)	41,806 (36.0)
Highest	93,786 (21.5)	9,317 (26.4)	75,282 (21.7)	24,852 (21.4)

**Table 3:** Adjusted RRs for Prenatal Care Outcomes Associated With County-Level Pregnancy Criminalization Rates in the 2 Years Before Birth, Overall and Stratified by Race and Ethnicity, Alabama, January 1, 2014–May 31, 2022

Pregnancy-Related Arrests Per 100,000 Births	Late or No Prenatal Care aRR (95% CI)				Inadequate Prenatal Care Utilization aRR (95% CI)			
	Overall	Black Births	White Births	Latina Births	Overall	Black Births	White Births	Latina Births

None	Ref.	Ref.	Ref.	Ref.	Ref.	Ref.	Ref.	Ref.
Medium	1.06 [1.04, 1.09]***	.95 [.91, 1.00]	1.05 [1.00, 1.10]	1.18 [1.12, 1.24]***	1.05 [1.03, 1.06]***	1.01 [.98, 1.03]	1.08 [1.06, 1.10]***	1.02 [.99, 1.06]
Highest	1.11 [1.08, 1.14]***	.97 [.91, 1.03]	1.08 [1.04, 1.13]***	1.18 [1.13, 1.24]***	1.01 [.99, 1.02]	.93 [.90, .96]***	1.04 [1.02, 1.06]***	.97 [.93, .99]*

*Abbreviations:* aRR, adjusted risk ratio; CI, confidence interval.

Note:

* *p* < .05

** *p* < .01

*** *p* < .001.
