# Supplementary material for: County-Level Pregnancy Criminalization Rates and Prenatal Care Initiation and Utilization in Alabama, 2014–2022
Source: Womens Health Issues. Author manuscript; Available in PMC 2026 Jun 10. (PMC13248919; doi:10.1016/j.whi.2026.03.004)
Supplement: 1 [file NIHMS2157272-supplement-Supplementary_Material.pdf]

APPENDIX

County-level pregnancy criminalization rates and prenatal care initiation and utilization in Alabama, 2014-2022

Taylor Riley, PhD MPH, Jaquelyn L. Jahn, PhD, MPH, Maeve Wallace, PhD, MPH

Sensitivity Analysis 1: Models adjusting only for individual factors

Appendix Table 1. Adjusted risk ratios (RR) for prenatal care outcomes associated with county-level pregnancy criminalization arrest rates, only adjusting for individual factors, overall and stratified by race and ethnicity, Alabama, January 1, 2014 – May 31, 2022

	Late or no prenatal care aRR (95% CI)				*Inadequate prenatal care utilization aRR (95% CI)*			
Pregnancy-related arrests per 100,000 births	***Overall***	***Black births***	***White births***	***Latina births***	***Overall***	***Black births***	***White births***	***Latina births***
None	Ref.	Ref.	Ref.	Ref.	Ref.	Ref.	Ref.	Ref.
Medium	0.99 (0.96, 1.02)	0.88 (0.84, 0.92)***	1.02 (0.98, 1.06)	1.01 (0.96, 1.06)	1.02 (1.01, 1.04)***	0.95 (0.93, 0.97)***	1.09 (1.07, 1.11)***	0.96 (0.93, 0.99)*
Highest	1.19 (1.16, 1.22)***	1.02 (0.96, 1.08)	1.15 (1.11, 1.20)***	1.29 (1.24, 1.35)***	1.01 (0.99, 1.02)	0.93 (0.90, 0.96)***	1.04 (1.02, 1.06)***	0.98 (0.95, 1.01)

County-level pregnancy criminalization rates were calculated based on the two years before birth. Models only adjusted for individual confounders and excluded county-level confounders (index of concentration at the extremes, OBGYN availability, jail incarceration rate)
aRR = adjusted risk ratio, CI = confidence interval
*p < .05, **p < .01, ***p <.001

Sensitivity Analysis 2:
Models additionally adjusting for county-level Latina population

Appendix Table 2. Adjusted risk ratios (RR) for prenatal care outcomes associated with county-level pregnancy criminalization arrest rates, additionally adjusting for county-level Latina population, Alabama, January 1, 2014 – May 31, 2022

	Latina births	
	Late or no prenatal care aRR (95% CI)	***Inadequate prenatal care utilization aRR (95% CI)***
Pregnancy-related arrests in the year before birth per 100,000 births		
None	Ref.	Ref.
Medium	1.07 (1.01, 1.14)*	1.02 (0.98, 1.06)
Highest	1.09 (1.04, 1.13)***	0.975 (0.94, 1.01)
Pregnancy-related arrests in the two years before birth per 100,000 births		
None	Ref.	Ref.
Medium	1.06 (1.003, 1.12)*	1.01 (0.98, 1.05)
Highest	1.03 (0.99, 1.08)	0.95 (0.91, 0.98)**

Sensitivity analysis restricted to births among Latina women.
aRR = adjusted risk ratio, CI = confidence interval
*p < .05, **p < .01, ***p <.001

Sensitivity Analysis 3:
Models include county-level pregnancy criminalization rates in the year before birth

Appendix Table 3. Adjusted risk ratios (RR) for prenatal care outcomes associated with county-level pregnancy criminalization arrest rates in the year before birth, overall and stratified by race and ethnicity, Alabama, January 1, 2014 – May 31, 2022

	Late or no prenatal care aRR (95% CI)				***Inadequate prenatal care utilization aRR (95% CI)***			
Pregnancy-related arrests in the year before birth per 100,000 births	***Overall***	***Black births***	***White births***	***Latina births***	***Overall***	***Black births***	***White births***	***Latina births***
None	Ref.	Ref.	Ref.	Ref.	Ref.	Ref.	Ref.	Ref.
Medium	1.10 (1.06, 1.13***	1.00 (0.95, 1.06)	1.12 (1.06, 1.18)***	1.14 (1.07, 1.21)***	1.06 (1.04, 1.08)***	1.02 (0.99, 1.05)	1.11 (1.08, 1.13)***	1.03 (0.99, 1.07)
Highest	1.12 (1.09, 1.15)***	1.01 (0.95, 1.07)	1.09 (1.05, 1.14)***	1.18 (1.13, 1.23)***	1.00 (0.99, 1.02)	0.95 (0.92, 0.97)**	1.03 (1.01, 1.05)*	0.99 (0.96, 1.02)

aRR = adjusted risk ratio, CI = confidence interval
*p < .05, **p < .01, ***p <.001

Sensitivity Analysis 4:
Models restricted to Alabama counties with documented arrests

Appendix Table 4. Adjusted risk ratios (RR) for prenatal care outcomes associated with county-level pregnancy criminalization arrest rates, overall and stratified by race and ethnicity, among Alabama counties with documented arrests, January 1, 2014 – May 31, 2022

	Late or no prenatal care aRR (95% CI)				***Inadequate prenatal care utilization aRR (95% CI)***			
Pregnancy-related arrests per 100,000 births	***Overall***	***Black births***	***White births***	***Latina births***	***Overall***	***Black births***	***White births***	***Latina births***
None	Ref.	Ref.	Ref.	Ref.	Ref.	Ref.	Ref.	Ref.
Medium	1.06 (1.03, 1.09)***	0.96 (0.91, 1.01)	1.05 (1.01, 1.10)*	1.10 (1.04, 1.16)***	1.05 (1.04, 1.07)***	1.02 (0.99, 1.04)	1.08 (1.06, 1.11)***	1.01 (0.98, 1.05)
Highest	1.09 (1.06, 1.12)***	0.97 (0.91, 1.04)	1.07 (1.03, 1.11)**	1.09 (1.04, 1.14)**	1.02 (1.002, 1.03)*	0.94 (0.91, 0.97)***	1.06 (1.04, 1.08)***	0.95 (0.92, 0.98)**

Models only among Alabama counties with any documented arrests during the study period. County-level pregnancy criminalization rates are calculated based on the two years before birth.
aRR = adjusted risk ratio, CI = confidence interval
*p < .05, **p < .01, ***p <.001
